# Characterizing clinical progression in patients with musculoskeletal pain by pain severity and central sensitization-related symptoms

**DOI:** 10.1038/s41598-024-55290-4

**Published:** 2024-02-28

**Authors:** Hayato Shigetoh, Masayuki Koga, Yoichi Tanaka, Yoshiyuki Hirakawa, Shu Morioka

**Affiliations:** 1 Department of Physical Therapy, Faculty of Health Science, Kyoto Tachibana University, Kyoto, 607-8175 Japan; 2 Neurorehabilitation Research Center, Kio University, Nara, 635-0832 Japan; 3 Department of Neurorehabilitation, Graduate School of Health Sciences, Kio University, Nara, 635-0832 Japan

**Keywords:** Pain, Central sensitization-related symptoms, Progress, Medical research, Signs and symptoms

## Abstract

Central sensitization-related symptoms (CSS) are associated with the severity and progression of pain. The relationship between the severity of pain/CSS and clinical progresses remains unclear. This multicenter, collaborative, longitudinal study aimed to characterize the clinical outcomes of patients with musculoskeletal pain by classifying subgroups based on the severity of pain/CSS and examining changes in subgroups over time. We measured the pain intensity, CSS, catastrophic thinking, and body perception disturbance in 435 patients with musculoskeletal pain. Reevaluation of patients after one month included 166 patients for pain intensity outcome and 110 for both pain intensity and CSS outcome analysis. We classified the patients into four groups (mild pain/CSS, severe pain/mild CSS, severe pain/CSS, and mild pain/severe CSS groups) and performed multiple comparison analyses to reveal the differences between the CSS severity groups. Additionally, we performed the adjusted residual chi-square to identify the number of patients with pain improvement, group transition, changing pain, and CSS pattern groups at baseline. The most characteristic result was that the mild and severe CSS groups showed worsening pain. Moreover, many of the group transitions were to the same group, with a few transitioning to a group with mild pain/CSS. Our findings suggest that the severity and improvement of CSS influence pain prognosis.

## Introduction

Many people experience pain and receive pain management, including medication, surgery, and rehabilitation, and most of their pain progress tends to recover^1,2^. However, pain progress is either maintained or worsened^3,4^; the diversity of pain processes reveals the complexity of pain pathology. Pain is classified as nociceptive, neuropathic, and nociplastic pain, and management based on the mechanism of each classification is essential^5^. Pain is caused by direct injury, psychological factors^6^, and body perception disturbance^7^ that influence and induce sensitization. The International Association for the Study of Pain (IASP) defined nociplastic pain as the “pain that arises from altered nociception despite no clear evidence of actual or threatened tissue damage causing the activation of peripheral nociceptors or evidence for disease or lesion of the somatosensory system causing the pain”^8^. An algorithm for nociplastic pain diagnosis has been proposed, and central sensitization-related symptoms (CSS) (including sleep disturbance and fatigue) are also considered important factors in the differentiation of nociplastic pain^9^.

CSS has been assessed using the central sensitization inventory (CSI) questionnaire and reported to be associated with pain^10^. CSI has been shown to be associated with pain severity^11^, and higher CSI scores have been reported to have a poorer prognosis^12^. The CSI score is important not only for the initial score but also for monitoring the amount of change, which is related to the prognostic value of pain^13^. However, CSI is not necessarily associated with pain severity, and there is a subgroup of patients with low pain but severe CSI^14^. Moreover, CSI is stratified and classified by severity according to scores, and subgroups have been reported in combination with pain severity^12,15^. However, the transition of subgroups from CSI to pain severity and the factors of the recovery process that are associated with the transition of subgroups remains unclear.

Comprehensive associations between the severity of CSS, process of CSS change, and pain process remain unknown. In addition, in the subgroup with severe CSS, it is unclear whether pain-related factors, such as negative psychological factors and body perception disturbances, are more severe. Consequently, we proposed three hypotheses as follows: (1) The subgroup with severe CSS is associated with severe negative psychological factors and body perception disturbances. (2) The amount of change in CSS is associated with the amount of change in pain. If the CSS in a subgroup with mild pain/severe CSS does not improve, the pain will not improve, and moving to a subgroup where both pain and CSS are severe. (3) The subgroup with mild CSS and severe pain is less affected by central sensitization. Therefore, chronic pain is less likely to occur, showing a trend toward pain improvement. This study aimed to characterize the clinical outcomes of patients with musculoskeletal pain by classifying subgroups based on the severity of pain and CSS and examining the characteristics of the subgroups through cross-sectional analysis and changes in subgroups over time.

## Results

### Comparison of four groups based on pain and CSS

**Figure 1:**
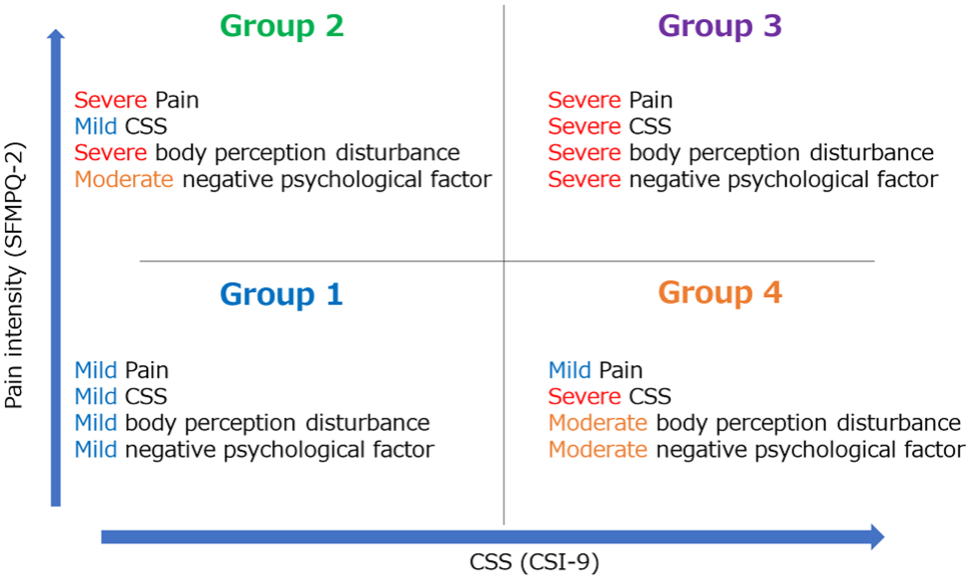
Characteristics of each group based on the results of multiple comparisons. Pain intensity (SFMPQ-2) on the vertical axis and CSS on the horizontal axis were used to summarize and illustrate the characteristics of the four groups based on severity. CSS, Central Sensitization-related Symptoms; SFMPQ-2, Short-form McGill Pain Questionnaire-2.

Table 1 shows the summary of the characteristics of the patients. All patients were classified into group 1 (mild pain/CSS), group 2 (severe pain/mild CSS), group 3 (Severe pain/CSS), and group 4 (mild pain/severe CSS) based on CSI-9 severity (≤ mild, severe ≤) and Short-form McGill Pain Questionnaire-2 (SFMPQ-2) z-score (< 0 / 0 ≤). The chi-square and Kruskal–Wallis tests showed significant differences in age, sex, and pain-related parameters (Numerical Rating Scale (NRS), Pain Catastrophizing Scale-6 (PCS-6), and Fremantle score). Residual analysis showed that patients in groups 1 and 2 were older than those in groups 3 and 4. In addition, group 4 had more female patients, and group 2 had fewer patients than other groups. Multiple comparisons showed that NRS scores were lower in groups 1 and 4 than in groups 2 and 3. The PCS-6 score was lower in group 1 than in groups 2, 3, and 4, and that of group 3 was higher than that of group 4. The Fremantle score was higher in group 3, followed by groups 2, 4, and 1, and there was no significant difference between groups 2 and 3 (Table 1). Figure 1 shows the characteristics of each group based on these results.

**Table 1:** Characteristics of patients in each group.

Variable	Group 1 (mild pain/ CSS) (n = 156)	Group 2 (severe pain/ mild CSS) (n = 52)	Group 3 (severe pain/ CSS) (n = 100)	Group 4 (mild pain/ severe CSS) (n = 127)	P-value
Age (years)	71.3 ± 13.4	72.3 ± 11.8	65.4 ± 18.1	65.3 ± 13.7	< 0.001
Sex	Male: 55, Female: 101	Male: 35, Female: 17	Male: 34, Female: 66	Male: 93, Female: 34	0.006
Acute/Chronic	63/93	17/35	36/64	34/93	0.12
SFMPQ-2	12.8 ± 9.1^a,b^	63.2 ± 33.3^a,e,^	72.0 ± 34.6^b,f^	18.6 ± 9.4^e,f^	< 0.001
CSI-9	4.8 ± 2.8^b,c^	4.9 ± 1.7^d,e^	15.8 ± 4.2^b,d^	14.8 ± 5.1^c,e^	< 0.001
NRS	4.8 ± 2.3^a,b^	6.0 ± 2.3^a,e^	7.0 ± 1.8^b,f^	5.0 ± 2.0^e,f^	< 0.001
PCS-6	7.5 ± 5.8^a,b,c^	13.0 ± 5.9^a^	15.3 ± 5.2^b,f^	11.0 ± 6.1^c,f^	< 0.001
Fremantle score	8.2 ± 5.8^a,b,c^	15.9 ± 8.0^a,e^	16.2 ± 6.9^b,f^	12.0 ± 7.1^c,e,f^	< 0.001

^“a”^ indicates a significant difference between groups 1 and 2, ^“b”^ indicates a significant difference between groups 1 and 3. ^“c”^ indicates a significant difference between group 1 and group 4. ^“d”^ indicates a significant difference between groups 2 and 3. ^“e”^ indicates a significant difference between groups 2 and 4. ^“f”^ indicates a significant difference between groups 3 and 4. CSS, central sensitization-related symptoms; SFMPQ-2, short-form McGill Pain Questionnaire-2; CSI-9, Central Sensitization Inventory-9; NRS, numerical rating scale; PCS-6, pain catastrophizing scale.

### Longitudinal progress of each group

The chi-square test results showed significant differences in the NRS score improvement rates between the groups, and residual analysis showed that only group 1 had a significantly higher NRS improvement rate (Table 2).

**Table 2:** Minimum clinically meaningful improvement achievers for pain in each group.

Variable	Group 1 (mild pain/ CSS) (n = 49)	Group 2 (severe pain/ mild CSS) (n = 15)	Group 3 (severe pain/ CSS) (n = 46)	Group 4 (mild pain/ severe CSS) (n = 56)	P-value
NRS score Improvement/ No improvement (% improvement achieve)	38/11 (77.6%)	10/5 (66.7%)	24/22 (52.2%)	30/26 (53.4%)	0.034
The adjusted residual Improvement/ No improvement	2.76*/−2.76*	−0.44/0.44	−1.52/1.52	−1.49/1.49	

“*” indicates a significant difference between the groups. CSS, Central Sensitization-related Symptoms; NRS, Numerical Rating Scale.

Table 3 shows the number of patients for the transition groups in the longitudinal progression. In the residual analysis, the number of patients in group 1 who remained there was significantly higher, and the number of patients who translated to group 3 was significantly lower. The number of patients in group 2 who remained in the same group was significantly higher. Moreover, the number of patients in group 3 who remained there was significantly higher, and the number of patients who translated to group 1 was significantly lower. The number of patients in group 4 who remained in the group was significantly higher, and the number of patients who translated to groups 1 was significantly lower.

**Table 3:** Adjusted chi-square residuals for the significant difference of transition between groups in the longitudinal.

Variable	Group 1 (mild pain/ CSS) (n = 32)	Group 2 (severe pain/ mild CSS) (n = 8)	Group 3 (severe pain/ CSS) (n = 30)	Group 4 (mild pain/ severe CSS) (n = 40)
Post group 1	30 (6.12*)	6 (1.58)	3 (−4.91*)	14 (−2.09*)
Post group 2	0 (−1.12)	2 (4.01*)	1 (−0.24)	0 (−1.33)
Post group 3	0 (−2.87*)	0 (−1.26)	12 (4.36*)	5 (−0.65)
Post group 4	2 (−3.89*)	0 (−2.09*)	14 (1.77)	21 (3.17*)

“*” indicates a significant difference between the groups. CSS: Central Sensitization-related Symptoms.

Focusing on the pattern of change in pain and CSS over longitudinal progress in the residual analysis, the patients whose pain increased significantly were those in group 4 (Table 4). Moreover, the number of patients whose pain increased was significantly lower in group 3.

**Table 4:** Adjusted chi-square residuals for the significant difference in change patterns of pain and CSS among groups.

Variable	Group 1 (mild pain/ CSS) (n = 32)	Group 2 (severe pain/ mild CSS) (n = 8)	Group 3 (severe pain/ CSS) (n = 30)	Group 4 (mild pain/ severe CSS) (n = 40)
Pain decrease/CSS decrease	15 (−1.67)	5 (0.20)	22 (1.86)	23 (−0.26)
Pain increase/CSS decrease	4 (0.14)	1 (0.06)	0 (−2.35*)	8 (2.01*)
Pain increase/CSS increase	4 (0.56)	0 (−0.98)	0 (−2.14*)	7 (1.98*)
Pain decrease/CSS increase	9 (−1.54)	2 (0.44)	8 (1.24)	2 (−2.84*)

“*” indicates a significant difference between groups. CSS: Central Sensitization-related Symptoms.

We calculated the statistical power focused on the main analysis (the chi-square test in the NRS score improvement rates between the groups) in this study. The statistical power was 0.70, using effect size (w = 0.23), sample size (n = 166), and the degree of freedom.

## Discussion

We used subgroup classification based on the severity of pain and CSS and adjusted chi-square residuals to characterize the clinical outcomes of patients with musculoskeletal pain and examined changes in subgroups over time. The results demonstrated that the severe pain/CSS group had the most severe catastrophic thoughts and body perception disturbance. This group also had the fewest transitioners to a group in which both pain and CSS were mild in the longitudinal change. In addition, the severe pain/mild CSS group had severe body perception disturbances, and the mild pain/severe CSS group had moderate catastrophic thoughts and body perception disturbances. Moreover, the mild pain/severe CSS group had more transitioners with worse pain in longitudinal change. On the other hand, the mild pain/CSS group had a higher number of pain improvement transitioners in the longitudinal change.

One aim of this study was to investigate the characteristic of subgroup based on the severity of pain and CSS, and this study showed that more women were in the mild pain/severe pain group and fewer women were in the severe pain/mild pain group. The severity of CSS in women has previously been demonstrated in studies of musculoskeletal pain patients and healthy individuals^16–18^ and has similar results to the present study. However, the severity of CSS in the severe pain/CSS group was not significantly higher than that in the other groups, although the proportion of females was higher (66%). CSS are caused by various stressors, including pain^19^. The severe pain/CSS group experienced the most pain and had high levels of catastrophic thinking and body perception disturbance, indicating that CSS is caused by more stress and not sex characteristics. Catastrophic thinking has been reported to be associated with pain intensity as a cognitive-emotional factor^11^, and cognitive-emotional sensitization^20^ may be responsible for increased pain intensity. Body perception disturbance has been reported to be associated with pain intensity^7^, and the factor of sensorimotor incongruence may be related to increased pain intensity as a pathological mechanism. One of the novel features of this study is the comparative validation of differences in the severity of negative psychological factors and body perception disturbances in subgroups based on pain and CSS severity.

Furthermore, this study investigated the changes in pain and CSS over time in subgroups based on pain and CSS. This is a unique point that can be emphasized as a novelty of this study. The results of the present study showed that the mild pain/CSS group with mild pain had significantly more patients with clinically meaningful pain improvement. Conversely, the groups with higher CSS (severe pain/CSS and mild pain/severe CSS groups) had more patients whose pain worsened or remained severe and those who had a poorer pain prognosis. The most common characteristic was severe CSS, which is included in the diagnosis algorithm for nociplastic pain^9^. Hypersensitivity to various sensory inputs is also included in the algorism of nociplastic pain diagnosis. Patients with nociplastic pain show activation of the default mode, frontoparietal, and attentional networks^21^ as well as activation of glial cells associated with pain neurotransmitters^22^. In addition, since the default mode, frontoparietal, and attentional networks are brain regions associated with cognitive-emotional and body perception, pain-related cognitive-emotional and body perception factors may be relevant to the pathological mechanism. Thus, the pathophysiology of poor pain prognosis in the severe CSS group in this study may be due to the sensitization state of the central nervous system, which exhibits sensory hypersensitivity to stimuli.

Our findings suggest that the pain prognosis is poor if the pain is mild but CSS is severe or if the change in CSS is poor. In contrast, there was also a group with mild CSS but severe pain, which was also shown to have severe body perception disturbance. A group with mild CSS/severe pain did not show any characteristic clinical changes in both pain and CSS. The changes in clinical outcomes in these subgroups may indicate that it is only possible to make favorable changes with interventions appropriate to the pathology. Interventions for nociplastic pain include cognitive behavioral therapy, education, and promoting good lifetime habits, including engaging in physical activity, weight management, sleep hygiene, and stress reduction^23^. In addition, interventions for body perception disturbances include graded motor imagery^24^. Since CSS is based on central nervous system pathology, it may be necessary to adapt and concentrate on interventions appropriate to central nervous system pathological mechanisms, such as cognitive functional therapy focusing on cognitive aspects^25^ and graded sensorimotor retraining focusing on body perception^26^. Notably, because various factors are associated with pain severity and poor prognosis, interventions based on pain pathophysiology may effectively improve pain. The stratification by subgroup classification and its characteristics were verified from multifaceted factors, including pain-related cognitive-emotional factors and body perception, and the prognosis of the stratified subgroups was an essential aspect of the novelty of this study.

This study has some limitations. First, the course and number of physical therapy sessions for each patient could not be controlled. Thus, the course of the drug therapy is unclear, and the differences in physiotherapy and drug therapy may have affected the outcomes after follow-up. However, all patients received weekly physical therapy, and since this study did not aim to determine the effectiveness of the intervention, it focused on pain and CSS change scores. Thus, the purpose of this study was achieved. Second, we could not determine the mechanisms underlying the differences in each group because neurotransmitter levels were not measured in this study. In addion, we could not measure the quantitative sensory testing (QST). QST is used as an indicator of central sensitization by measuring temporal summation to capture the wind-up phenomenon in the dorsal horn of the spinal cord, and conditioned pain modulation is used with the idea that it can capture the activity of the descending pain inhibitory system. Although both QST and CSI are used to capture the pathophysiology of central sensitization, it is assumed that they are assessing different aspects of the pathophysiology of central sensitization. Therefore, a limitation of this study is that it evaluates central sensitization in terms of the severity of central sensitization-related symptoms by CSI, which only explains some aspects of the pathogenesis of central sensitization. Third, this study could only examine the longitudinal course of CSS for one month. Some studies can assume that changes in CSS occur for one month^13^. Still, since some studies have found intervention effects over extended periods (3 or 6 months)^27^ Therefore, the low number of participants who changed the group classification might be related to the short follow-up period. Future studies should include long-term follow-up to further investigate the applicability of longitudinal changes. Fourth, not all cases that were analyzed cross-sectionally completed the longitudinal evaluation. Statistical power was 0.7, a constant statistical power, although not above the standard value of 0.8, which is considered good^28^. However, there was a risk of selection bias because this study was based on a cross-sectional analysis of subjects for whom changes over time could be confirmed. This also needs to be revised regarding the generalizability of the results of this study.

To the best of our knowledge, this is the first study to investigate the characteristics of the clinical outcomes of patients with musculoskeletal pain by classifying subgroups based on the severity of pain and CSS and examining changes in subgroups over time. The findings of this study suggest that the severity and improvement of CSS influence pain prognosis. Additionally, these findings may help tailor-made interventions to reduce pain.

## Methods

### Patients

The study was conducted between April 2021 and December 2022 and included 435 inpatients or outpatients (mean age 68.3 ± 14.8 years) who were recruited from multicenter clinics and hospitals. The inclusion criteria were patients who complained of pain (NRS score ≥ 1). The exclusion criteria were patients diagnosed with dementia or significantly higher brain dysfunction with difficulty answering the questionnaire. All patients (*n* = 435) completed questionnaires at baseline. All patients received the same category of physical therapy (manual therapy, exercise, and education), not including the specific concept intervention, such as cognitive behavioral therapy and graded motor imagery. One month later, a reevaluation was performed on the subjects. A total of 166 patients who were able to assess the pain intensity outcome overtime at the time of reassessment and 110 patients who could fully assess both the pain intensity and CSS outocome over time were included in the analysis. This study conformed to the principles of the Declaration of Helsinki. All participants provided written informed consent before the study commenced. This study was approved by the ethics committee of Kio University Health Sciences Graduate School (approval no. R3-04).

### Questionnaires

Patient demographic data (age, sex, pain duration (acute: < 3 months and chronic: ≤ 3 months), NRS, and SFMPQ-2 for pain intensity, shortened version of the CSI (CSI-9) for CSS, PCS-6 for pain catastrophizing, and Fremantle score for body perception disturbance, were collected.

The CSI-9 questionnaire is a simplified form of the Japanese version of the CSI, and its reliability and validity have been previously confirmed^29^. The CSI-9 comprises nine CSS items, and each item is evaluated between 0 (none) and 4 (always), with a total score ranging from 0 to 36. The total score is classified into three severities: subclinical (0–9), mild (10–19), and moderate/severe (20–36)^29^.

The NRS for pain was used to assess pain intensity (0, no pain; 10, worst pain imaginable). In patients with musculoskeletal pain, an improvement of more than "22% (acute pain)" or "33% (chronic pain)" was considered to be a minimally clinically important difference (MCID)^30,31^.

The SFMPQ-2 was used to assess pain intensity and included items that assessed 22 qualities of pain and the intensity of each quality on an 11-point NRS^32^, and a higher score indicated worse pain and vice versa. The SFMPQ-2 had four subclasses: one affective and three sensory (continuous pain, intermittent pain, and neuropathic pain) subclasses.

PCS-6 was used to assess pain catastrophizing. The PCS-6 is a shorter version of the 13-item PCS, contains six items, and has good internal consistency and construct validity^33^. Notably, higher scores indicated more severe catastrophic thinking and vice versa.

The Fremantle score was used to assess disturbed body perception. The back pain section of the Fremantle back awareness questionnaire^7^ was changed to the pain area, and higher scores indicated more severe body perception disturbances.

### Statistical analysis

Our main analysis examined the change in pain improvement/non-improvement in four groups of patients. Based on the 4 × 2 chi-square test, a sample size of 122 is required to detect a medium-sized effect with a power of 0.80 and a degree of freedom of 3.

The SFMPQ-2 (pain) and CSI-9 (CSS) scores were used to classify the groups based on pain and CSS. Although the NRS also assesses pain, it was decided that the SFMPQ-2, which scores a wider range of descriptions, was more appropriate for group classification than simple pain intensity. Based on the standardized SFMPQ-2 score (z-score) of all patients, pain was defined as mild (z-score < 0) or severe (z-score ≥ 0)^34^. In addition, based on the severity classification of CSI-9, CSS was defined as mild CSS (CSI-9 score ≤ 19) or severe CSS (CSI-9 score 20 ≤)^29^. The patients were classified into four groups according to the following criteria: mild pain/CSS (group 1), severe pain/mild CSS (group 2), severe pain/CSS (group 3), and mild pain/severe CSS (group 4).

In the first analysis as a cross-sectional analysis, demographic data (age, sex, pain duration), SFMPQ-2, CSI-9, NRS, PCS-6, and Fremantle scores were compared between the groups. The Kruskal–Wallis test was used to analyze continuous variables. Multiple comparisons were performed using Tukey’s method with Bonferroni correction. The significance level was set at < 5%.

In the second analysis as a longitudinal analysis, we identified the number of patients with pain improvement during longitudinal progress based on the MCID of the NRS (acute pain; ≥ 22%^30^, chronic pain; ≥ 33%^31^). We calculated the adjusted chi-square residuals considering 1.96 as the critical value (α = 0.05). Adjusted residuals can be used to determine the significant contributor to a significant chi-squared result.

In the third analysis as a longitudinal analysis, we also identified the number of patients with group transition at post 1 month and the number of patients with changing pain and CSS pattern group (increase or decrease pain/CSS). We calculated the adjusted chi-square residuals considering 1.96 as the critical value (α = 0.05). Adjusted residuals can be used to determine the significant contributor to a significant chi-squared result. Statistical analyses were performed using R software (ver. 4.1.2).

## Data Availability

The datasets used and/or analysed during the current study available from the corresponding author on reasonable request.
